# Sirtuins 1 and 2 in the Acute Period After Photothrombotic Stroke: Expression, Localization and Involvement in Apoptosis

**DOI:** 10.3389/fphys.2022.782684

**Published:** 2022-04-27

**Authors:** Moez Eid, Valentina Dzreyan, Svetlana Demyanenko

**Affiliations:** Laboratory of Molecular Neurobiology, Academy of Biology and Biotechnology, Southern Federal University, Rostov-on-Don, Russia

**Keywords:** histone deacetylases, sirtuins, photothrombotic stroke, penumbra, apoptosis

## Abstract

Sirtuins (SIRTs) are NAD^+^- dependent histone deacetylases. They are involved in a variety of biological pathways and are thought to be a promising target for treating several human disorders. Although evidence is piling up to support the neuroprotective role of SIRTs in ischemic stroke, the role of different sirtuin isoforms needs further investigation. We studied the effects of photothrombotic stroke (PTS) on the expression and localization of sirtuins SIRT1 and SIRT2 in neurons and astrocytes of the penumbra and tested the activity of their selective and non-selective inhibitors. SIRT1 levels significantly decreased in the penumbra cells nuclei and increased in their cytoplasm. This indicated a redistribution of SIRT1 from the nucleus to the cytoplasm after PTS. The expression and intracellular distribution of SIRT1 were also observed in astrocytes. Photothrombotic stroke caused a sharp increase in SIRT2 levels in the cytoplasmic fraction of the penumbra neurons. SIRT2 was not expressed in the penumbra astrocytes. SIRT1 and SIRT2 did not co-localize with TUNEL-positive apoptotic cells. Mice were injected with EX-527, a selective SIRT1 inhibitor; SirReal2, selective SIRT2 inhibitor or salermide, a nonspecific inhibitor of SIRT1 and SIRT2. These inhibitors did not demonstrate any change in the infarction volume or the apoptotic index, compared to the control samples. The studies presented indicate the involvement of these sirtuins in the response of brain cells to ischemia in the first 24 h, but the alterations in their expression and change in the localization of SIRT1 are not related to the regulation of penumbra cell apoptosis in the acute period after PTS.

## Introduction

Stroke is one of the leading causes of mortality globally and a major cause of disability (Cushman et al., 2008; Donnan et al., 2008; Katan and Luft, 2018).

Stroke claimed the lives of 6.6 million people worldwide in 2019. Half of them died of ischemic stroke (Virani et al., 2021).

Vascular recanalization therapies (including tissue plasminogen activator thrombolysis and thromboembolectomy) are now the most effective treatments, although they are limited by a short therapeutic window and various safety issues (Del Zoppo et al., 2009; Albers et al., 2018; Nogueira et al., 2018; Thomalla et al., 2018).

The ineffectiveness of current therapies suggests that there must be additional important mechanisms underlying the pathophysiology of ischemic stroke (Zhang et al., 2018).

Sirtuins, or silent information regulator 2 (SIR2) proteins, are class III nicotine adenine dinucleotide (NAD^+^) - dependent histone deacetylases. Seven isoforms of sirtuins have been identified in mammals (SIRT1-SIRT7) (Michishita et al., 2005; Khoury et al., 2018).

SIRT1 and SIRT6 are mainly located in the nucleus of cells, SIRT7 in the nucleoli, SIRT2 is usually found in the cytoplasm, and SIRT3, SIRT4, and SIRT5 are mitochondrial proteins. Sirtuins deacetylate a variety of substrates, including histones, transcription factors, and cellular metabolic and cytoskeletal enzymes. They control metabolism, cell growth, responses to damaging influences, apoptosis, and autophagy (Michishita et al., 2005; She et al., 2017; Khoury et al., 2018).

Our work is devoted to studying the role of non-mitochondrial sirtuins SIRT1 and SIRT2 in brain cells after ischemic stroke.

SIRT1 is the most extensively studied sirtuin in mammals. Most publications indicate the ability of SIRT1 to increase neuronal survival (Petegnief and Planas, 2013; Khoury et al., 2018). Administration of SIRT1 activators, for example, resveratrol, reduces oxidative stress, neuronal apoptosis, and decreases the level of proinflammatory cytokines in stroke models (Petegnief and Planas, 2013; Yang et al., 2013). Overexpression of SIRT1 *via* viral vectors promotes post-ischemic recovery (Yan et al., 2013). SIRT2 is a predominantly cytoplasmic protein. Its substrates are proteins involved in the regulation of transcription, organization of the cytoskeleton, dynamics of microtubules, etc (North et al., 2003; Rauh et al., 2013). Unlike SIRT1, SIRT2 is more associated with the damage of neurons after ischemia.

An increase of SIRT2 in the cell nuclei was observed on the first day following the middle cerebral artery occlusion (MCAO). Suppression of SIRT2 expression had a neuroprotective effect (Xie et al., 2017). In cultured cells, an increase in the SIRT2 levels under oxidative stress led to apoptosis (Nie et al., 2014).

However, the ability of SIRT1 and SIRT2 to translocate between cell compartments in response to ischemia and participate in the acetylation of both nuclear and cytoplasmic proteins indicates a more complex role of these deacetylases in the pathogenesis of stroke. In addition, it is known that SIRT1 deacetylates its downstream targets, including NF-κB, Foxos, p53, PGC-1α, HIF, UCP, etc., in order to regulate the processes of oxidative stress, apoptosis, and inflammation during ischemia, both in the nuclei and in the cytoplasm of neurocytes (Meng et al., 2017; Yan et al., 2019). During brain development and in response to physiological and pathological stimuli, SIRT1 intracellular localization can change (Tanno et al., 2007; Hisahara et al., 2008). Furthermore, it is not yet clear how an increase in the expression of NAD^+^-dependent SIRT1 or stimulation of its activity for neuroprotection will affect the early stages of ischemia, when mitochondrial function is disrupted and a strong energy deficit is observed. Perhaps, on the contrary, the activity of SIRT1 should be blocked because it increases the depletion of energy in the cells of the penumbra? To answer these questions, more research is needed on the overexpression and/or suppression of SIRT1 and SIRT2.

In this work, we investigated the expression and intracellular localization of SIRT1 and SIRT2 in neurons and astrocytes of the peri-infarct area on the first day after photothrombotic stroke (PTS), as well as the effect of selective SIRT1 and SIRT2 inhibitors on the apoptosis and the damage volume in the peri-infarct area after PTS.

## Research Methods

### Animals

Adult male rats (200–250 g) were used as animal models. Experiments with enzyme inhibitors were performed on male mice (20–25 g, 14–15 weeks old) of the outbred stoke CD-1. The animals were kept under standard conditions (12-h light/dark cycle, open access to water and food at 22–25°C, an air exchange rate—18 shifts/hour). Body temperature was regularly measured using a rectal thermometer and maintained within 37 ± 0.5°C (using an electric mat). All international, national, and/or institutional guidelines for the care and use of animals were followed. All experimental techniques were performed in conformity with European Union standards 86/609/EEC for the use of experimental animals and local legislation for ethics of experiments on animals. The Southern Federal University’s Animal Care and Use Committee assessed and approved the animal protocols (Approval No. 08/2016). There was no special randomization used to assign subjects to the study.

### Photothrombotic Stroke

A diode laser irradiation (532 nm, 60 mW/cm^2^, 3 mm beam diameter, 30 min) was used to induce aphotothrombotic stroke (PTS) in a portion of the rat cerebral sensorimotor cortex following an intravenous injection of photosensitizer Bengal Rose (R4507, SigmaAldrich; 20 mg/kg) (Uzdensky et al., 2017; Uzdensky, 2018).

The same photosensitizer was injected intraperitoneally at a dose of 15 mg/ml (10 μl/g body weight) to induce PTS in the cerebral cortex of mice. This was followed by the irradiation of the sensorimotor cortex (2 mm lateral to the bregma) using a diode laser (532 nm, 200 mW/cm^2^, 1 mm beam diameter, 15 min) (Demyanenko et al., 2018, 2019).

Control: The identical procedures and operations were applied to sham-operated animals but without the introduction of a photosensitizer.

### Immunofluorescence Microscopy

Anesthesia of rats and transcardial perfusion with 10% formalin were performed 4 or 24 h after PTS. The brain was extracted and fixed overnight with formalin and incubated in 20% sucrose in Phosphate-buffered saline (PBS) for 48 h at 4°C. Using the vibratome Leica VT 1000 S (Germany), frontal sections (20 μm thickness) were obtained and then frozen in 2-methyl butane and stored at −80°C. After thawing, and washing with PBS, nonspecific antibody binding was blocked by using 5% bovine serum albumin (BSA) and 0.3% Triton X-100 (20–25°C, 1 h).

Sections were incubated with primary rabbit antibodies (SigmaAldrich): anti-Sirt1 (AV32386, 1:100), anti-Sirt2 (S8447, 1:500), anti-mouse antibodies-NeuN (MAB377; 1:1000),or anti-GFAP (SAB5201104; 1:1000) overnight at 4°C. Sections then were washed in PBS and incubated with fluorescently labeled secondary anti-rabbit antibodies CF488A (SAB4600045, 1:1000) or anti-mouse antibodies CF555 (SAB4600302, 1:1000) for 1 h. An Eclipse FN1 microscope (Nikon, Japan) was used to analyze the sections after being mounted in 60% glycerol in PBS. Control: Sections subjected to the same conditions, but without primary antibodies.

Fluorescence images of the central penumbra regions and peripheral areas were studied at a distance of 0.3–0.7 mm, 1.2–1.5 mm from the infarct core border, respectively. Quantitative assessment of fluorescence was performed using 10–15 images of experimental and control preparations acquired with the same camera settings. ImageJ software (http://rsb.info.nih.gov/ij/) was used to calculate the average fluorescence intensity in each image. To calculate the corrected total cell fluorescence intensity I (CTCF), proportional to the level of protein expression, we used the following equation:

I=Ii-Ac∗Ib;

Where Ii: integrated fluorescence intensity, Ac: cell area, Ib: average background fluorescence (Demyanenko et al., 2018). For all images, the threshold values remained constant. To calculate the relative changes in the fluorescence between the penumbra cells and the control cortex, ΔI, the following equation was used:

ΔI=(Ipen-Ic)⁄Ic;

Where Ipen: average fluorescence intensity in the penumbra, Ic: average fluorescence intensity in the control samples.

Protein co-localization with markers (neuron marker-NeuN, or astrocyte marker-GFAP) was studied and observed using ImageJ software with the JACoP plugin (Bolte and Cordelières, 2006). In RGB images, the Manders’ coefficient (M1) reflects the fractions of red signals (TUNEL staining) and green signals (proteins of interest) in the total signal registered in the red channel (Manders et al., 1992).

The change in SIRT1 localization in penumbra cells was assessed by determining its co-localization with the cell nucleus marker—Hoechst 33342 (10 μg/ml; blue signal) 4 or 24 h after PTS. Three visual fields were analyzed in each brain region of 6–8 rats. Statistical analysis was done according to One Way ANOVA. The obtained results are presented as M ± SEM.

### TUNEL Assay

TdT-mediated dUTP-X nick end labeling (TUNEL assay) was used to visualize apoptotic cells, which were detected (red signal) by using *In Situ* Cell Death Detection Kit, TMR red (# 12156792910, Roche). Brain sections were incubated at 37°C with primary antibody against the studied protein (green signal), then washed, subjected to cell detection kit, and incubated with the secondary antibody Anti-Rabbit CF488A (SAB4600045, 1:1000) and Hoechst 33342 (10 μg/ml, blue signal) for 1 h. The following equation was used to calculate the apoptotic index (AI):

AI = (TUNEL-positive cells/total Hoechst 33342-labeled cells)

Three images were analyzed for each of the seven animals in the group. The obtained results were processed by using One Way ANOVA and presented as M ± SEM.

### Western Blotting

Rats were euthanized with an overdose of chloral hydrate (600 mg/kg, intraperitoneally) at 4 or 24 h after PTS. Infarction core was removed on ice from the isolated brain by using a cylindrical knife (Ø 3 mm). Another knife (Ø 7 mm) was used to cut out the surrounding 2 mm ring, around the irradiation area, corresponding approximately to the penumbra tissue. The control sample was cut out from the non-irradiated contralateral hemisphere of sham-operated rats’ cerebral cortex.

Tissues were stored at −80°C after homogenization on ice, and freezing quickly in liquid nitrogen. Using the CelLytic NuCLEAR Extraction Kit (Sigma-Aldrich), cytoplasmic and nuclear fractions were isolated. The precipitate contained cellular nuclei, therefore represented the nuclear fraction, and the total supernatant was used as the cytoplasmic fraction.

Experiments included the use of primary rabbit antibodies (SigmaAldrich): anti-Sirt1 (AV32386, 1:500), anti-Sirt2 (S8447, 1:500), and mouse anti-β-actin antibody (A5441, 1:5000). Also secondary antibodies (SigmaAldrich): anti-Rabbit IgG-Peroxidase (A6154, 1:1000) and anti-Mouse IgG-Peroxidase (A4416, 1: 1000). Obtained results were processed by using One Way ANOVA and presented as M ± SEM.

### Inhibitors Administration

We studied the potential effects of EX-527 (E7034), a selective SIRT1 inhibitor (Lv et al., 2015; Fan et al., 2018; Zhu et al., 2019); SirReal2 (SML1514), a selective SIRT2 inhibitor; and salermide (S8825), a non-selective inhibitor of SIRT1 and SIRT2 (Alessandrini et al., 1999; Zhao et al., 2012). All inhibitors were administered 1 h after PTS.

The dose of SirReal2 or salermide was 400 μM. These inhibitors were dissolved in dimethyl sulfoxide (DMSO) and diluted in sterile saline. They were intracerebroventricularly administrated using a Hamilton injection syringe (bregma −0.9 mm lateral, −0.1 mm posterior, −3.1 mm deep). The injection had a volume of 0.2 ml, and it was administrated one time at 1 h after photothrombotic stroke. Control: sham-operated animals injected with DMSO dissolved in physiological saline.

The dose of the intraperitoneal injection of EX-527 was 5 mg/kg. The inhibitor was dissolved in sterile physiological saline and further administered in a volume of 0.2 ml once a day, for 7 days. Control animals were injected with sterile physiological saline.

### Evaluation of the Infarction Volume

The infarction volume was determined at different times after PTS. After the anesthesia and decapitation of mice, brains were isolated then placed into cooled brain matrices (J&K Seiko Electronic Co., Ltd.). These matrices were kept in −80°C for 3–5 min, then sliced (2 mm thickness). Sections were stained using 1% 2, 3, 5-triphenyl tetrazolium chloride (TTC; T8877, Sigma) in the dark at 37°C for 30 min. The infarctions zones in each section were analyzed using the ImageJ software (http://rsb.info.nih.gov/ij/).

### Statistical Analysis

One-Way ANOVA with Dunnett’s post hoc test was used for statistical analysis. All data were presented as Mean ± SEM. “N” indicates the number of animals in the experimental groups.

## Results

### SIRT1

**FIGURE 1:**
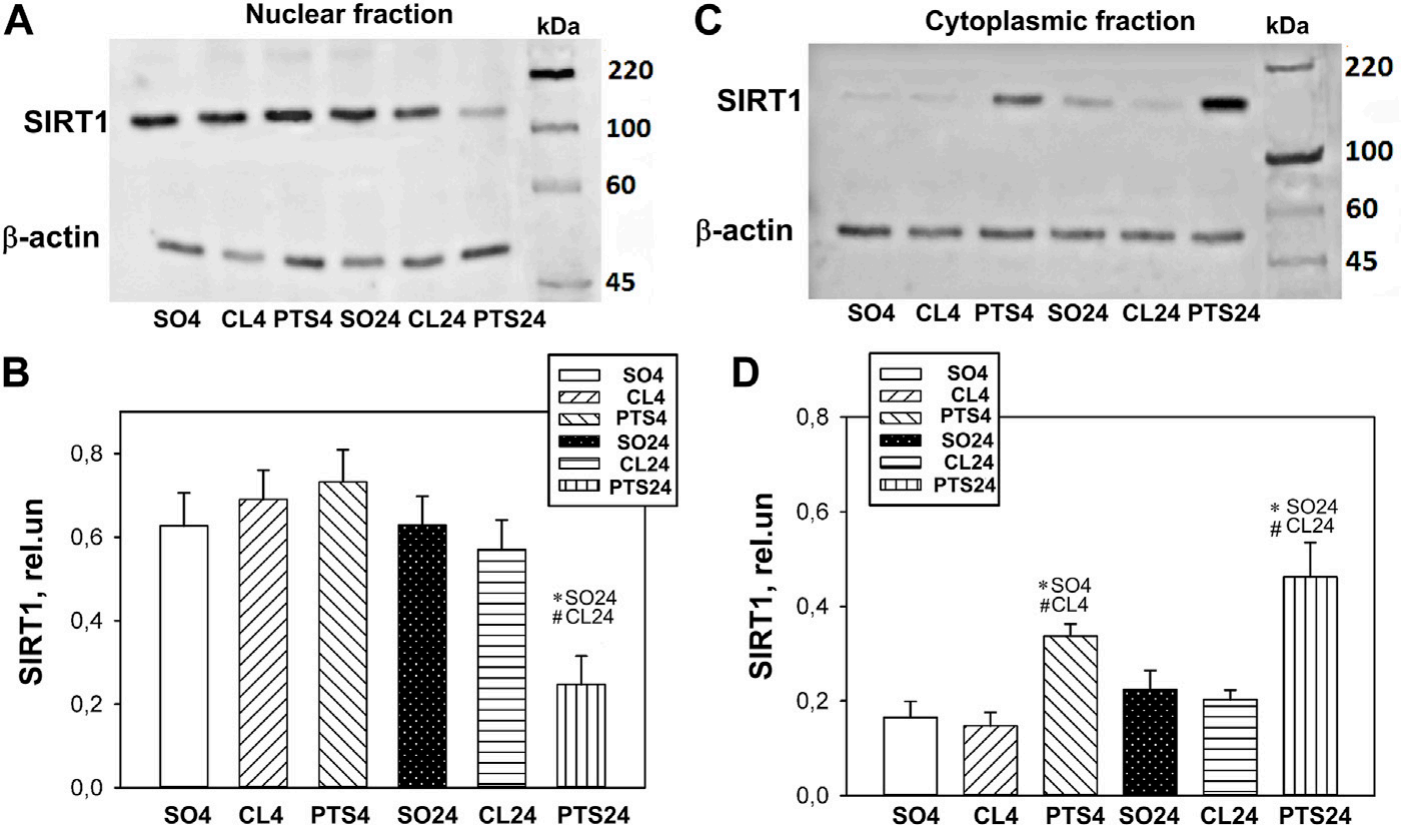
SIRT1 expression in nuclear **(A,B)** and cytoplasmic **(C,D)** fractions of penumbra tissue 4 or 24 h after photothrombotic stroke in the cerebral cortex of rats. One-way ANOVA. M ± SEM. *n* = 7. *p < 0.05 compared to sham-operated animals, #*p* < 0.05 compared to the contralateral cerebral cortex of the same animals.

According to Western blotting, it was shown that in control samples of the rat cerebral cortex, SIRT1 is localized mainly in the cell nuclei, and its level is significantly lower in the cytoplasm (Figure 1).

Twenty four hours after PTS, the level of SIRT1 in the penumbra nuclear fraction decreased by more than two times in comparison with both controls (*p* < 0.05) and increased in the cytoplasmic fraction (*p* < 0.05). Moreover, a significant increase in the level of SIRT1 in the cytoplasm was noted as early as 4 h after PTS (Figures 1C,D). This indicates a redistribution of SIRT1 from the nucleus to the cytoplasm of the penumbra cells.

**FIGURE 2:**
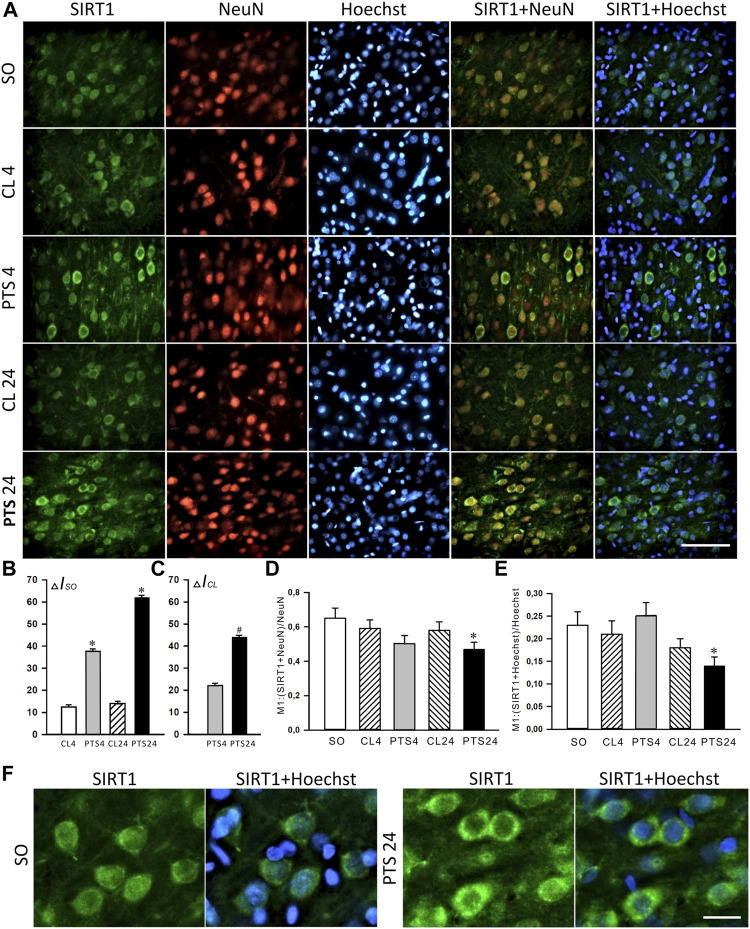
SIRT1 levels and localization in the neurons of ischemic penumbra 4 and 24 h after photothrombotic stroke in the rat cerebral cortex (PTS4 and PTS24, respectively) compared to the same animals’ contralateral cortex (CL4 and CL24), or the cerebral cortex of sham-operated animals (SO). **(A)** Immunofluorescence of SIRT1 (green), neuronal marker NeuN (red), nuclear chromatin marker Hoechst 33342 (blue), and image overlay. The scale bar is 100 μm. **(B)** Percent changes (ΔISO) of SIRT1 levels in the penumbra (PTS4 or PTS24) and the contralateral hemisphere (CL4 or CL24) compared to the brain cortex of the sham-operated rats (SO) 4 or 24 h after the PTS. **(C)** Percent changes (ΔICL) of SIRT1 levels in the penumbra (PTS4 or PTS24) compared to the contralateral cortex of the same rats 4 or 24 h after PTS. **(D)** Coefficient M1 of SIRT1 co-localization with the neuronal marker (NeuN) in different control and experimental groups. **(E)** Coefficient M1 of SIRT1 co-localization with the nuclear marker (Hoechst 33342) in different control and experimental groups. **(F)** Immunofluorescence of SIRT1 in the penumbra at bigger magnification (×40). The scale bar is 30 μm. One Way ANOVA; M ± SEM; *n* = 7.

Immunofluorescence microscopy also showed a significant increase in the level of SIRT1 in penumbra cells 4 and 24 h after PTS (Figures 2A,B). This increase was most noticeable in the cytoplasm of neurons (Figure 2A). The coefficients of SIRT1 co-localization with the neuronal marker NeuN (Figure 2D) and the cell nuclear marker Hoechst 33342 (Figure 2E) in the penumbra significantly decreased 24 h after PTS. These data also indicate the redistribution of SIRT1 from nuclei to the cytoplasm in penumbra cells after photothrombotic stroke. But since an increase in the level of SIRT1 in the cytoplasm was observed as early as 4 h after PTS, before the level in neuronal nuclei decreased, it can be assumed that at this time in the cytoplasm of neurons there was an intensive synthesis and accumulation of this protein.

**FIGURE 3:**
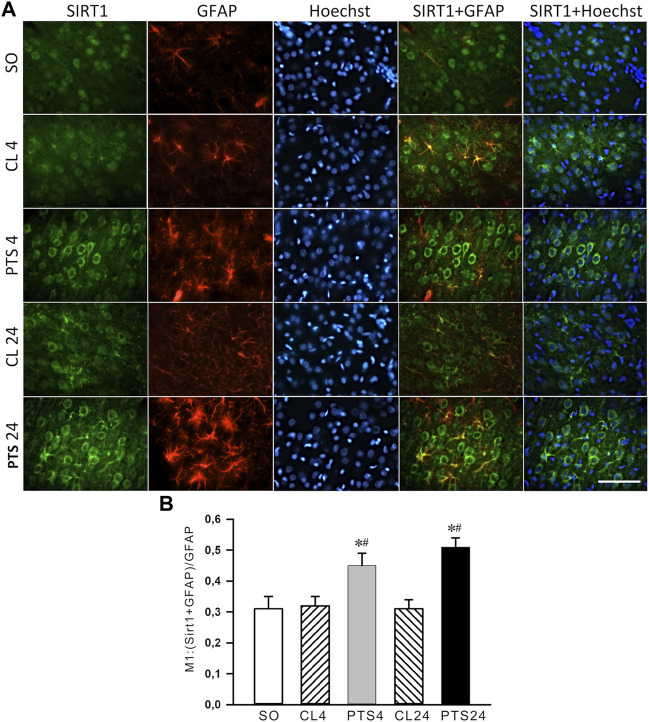
SIRT1 levels in the astrocytes of ischemic penumbra 4 and 24 h after photothrombotic stroke in the rat cerebral cortex (PTS4 and PTS24, respectively) compared to the same animals’ contralateral cortex (CL4 and CL24), or the cortex of sham-operated animals (SO). **(A)** Immunofluorescence of SIRT1 (green), marker of astrocytes GFAP (red), nuclear chromatin marker Hoechst 33342 (blue), and image overlay. The scale bar is 100 μm. **(B)** Coefficient M1 of SIRT1 co-localization with astrocyte marker (GFAP) in different control and experimental groups. One Way ANOVA; M ± SEM; *n* = 7.

The presence of SIRT1 was also noted in the nuclei of some astrocytes (Figure 3A). 4 and 24 h after PTS, the coefficient of SIRT1 co-localization with the astrocyte marker GFAP increased significantly (Figure 3B), and SIRT1 was also observed in the cytoplasm (Figure 3A). But since astrocytes containing SIRT1 are few in comparison with neurons (Figure 2A), the intracellular redistribution of SIRT1 in neurons was more significant.

### SIRT2

**FIGURE 4:**
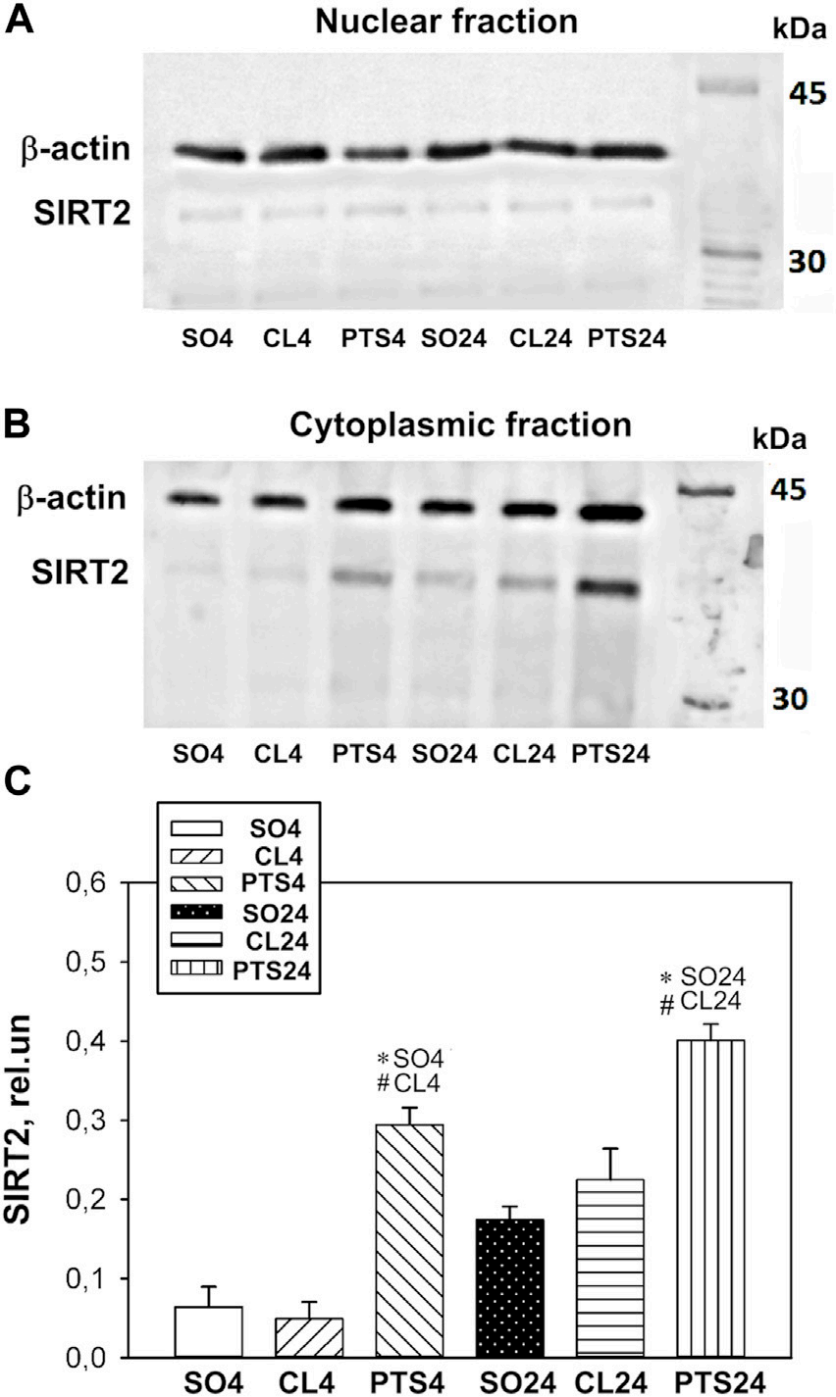
SIRT2 expression in nuclear **(A)** and cytoplasmic **(B,C)** fractions of penumbra tissue 4 or 24 h after photothrombotic stroke in the cerebral cortex of rats. One-way ANOVA. М ± SEM. *n* = 7. *p < 0.05 compared to sham-operated animals, #*p* < 0.05 compared to the contralateral cerebral cortex of the same animals.

In contrast to SIRT1, SIRT2 was localized mainly in the cytoplasmic fraction of the penumbra, and the nuclear fraction was practically not detected by Western blotting (Figure 4A).

**FIGURE 5:**
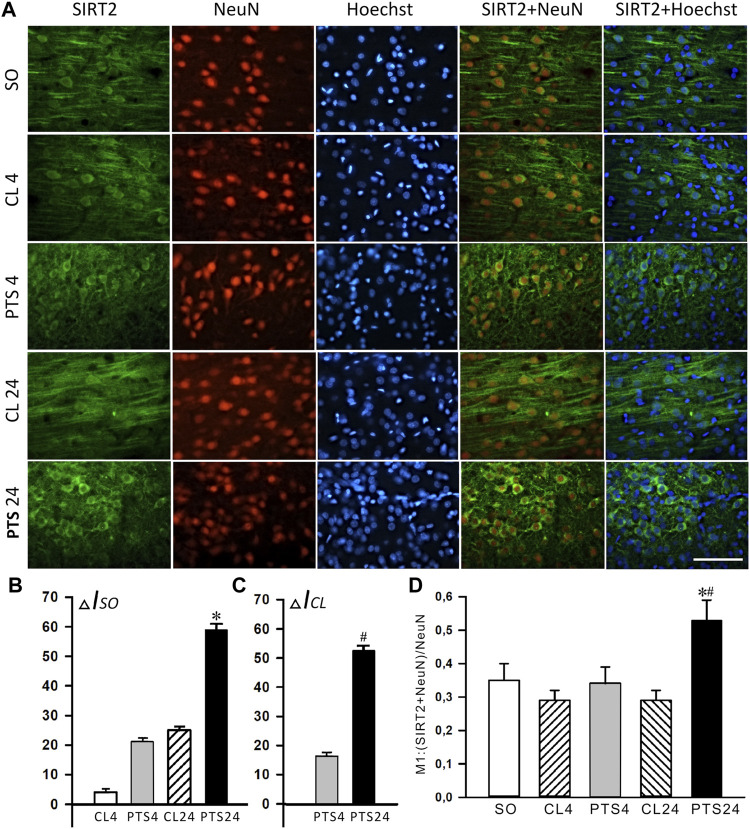
SIRT2 levels and localization in the neurons of ischemic penumbra 4 and 24 h after photothrombotic stroke in the rat cerebral cortex (PTS4 and PTS24, respectively) compared to the same animals’ contralateral cortex (CL4 and CL24), or the cortex of sham-operated animals (SO). **(A)** Immunofluorescence of SIRT2 (green), neuronal marker NeuN (red), nuclear chromatin marker Hoechst 33342 (blue), and image overlay. The scale bar is 100 μm. **(B)** Percent changes (ΔISO) of SIRT2 levels in the penumbra (PTS4 or PTS24) and the contralateral hemisphere (CL4 or CL24) compared to the brain cortex of sham-operated rats (SO) 4 or 24 h after PTS. **(C)** Percent changes (ΔICL) of SIRT2 levels in the penumbra (PTS4 or PTS24) compared to the contralateral cortex of the same rats 4 or 24 h after PTS. **(D)** Coefficient M1 of SIRT2 co-localization with the neuron marker (NeuN) in different control and experimental groups. One Way ANOVA; M ± SEM; *n* = 7.

Photothrombotic stroke caused a sharp increase in the level of SIRT2 in the cytoplasmic fraction of the penumbra after 4 and 24 h (Figures 4B,C). Immunofluorescence microscopy confirmed these data, but a significant increase in the SIRT2 level was observed only after 24 h (Figures 5A–C). At this time, the co-localization of SIRT2 with the marker of neuronal nuclei NeuN also increased (Figure 5D), which indicates the appearance of SIRT2 in the nuclei of neurons.

**FIGURE 6:**
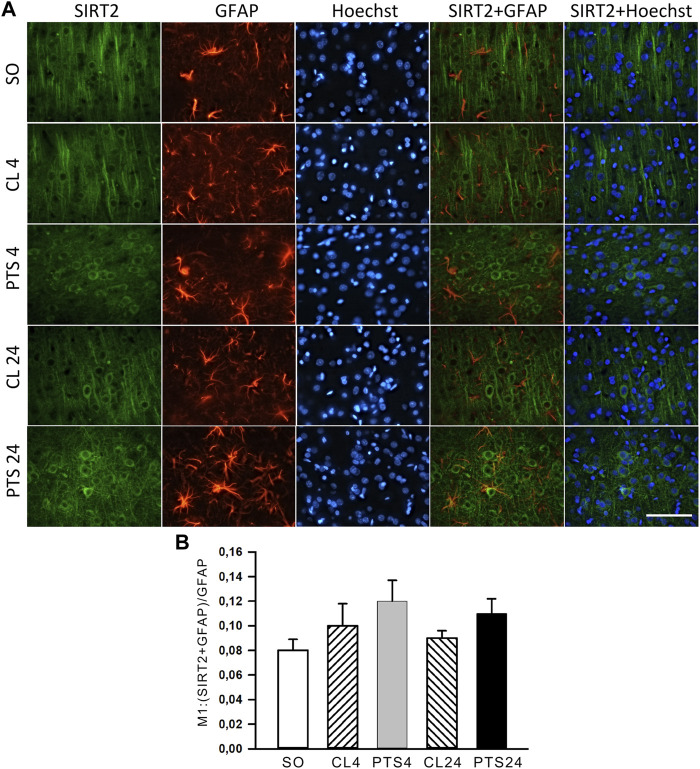
SIRT2 levels in astrocytes of ischemic penumbra 4 and 24 h after photothrombotic stroke in the rat cerebral cortex (PTS4 and PTS24, respectively) compared to the same animals’ contralateral cortex (CL4 and CL24), or the cortex of sham-operated animals (SO). **(A)** Immunofluorescence of SIRT2 (green), marker of astrocytes GFAP (red), nuclear chromatin marker Hoechst 33342 (blue), and image overlay. The scale bar is 100 μm. **(B)** Coefficient M1 of SIRT2 co-localization with the astrocyte marker GFAP in different control and experimental groups. One Way ANOVA; M ± SEM; *n* = 7.

SIRT2 was practically not expressed in penumbra astrocytes, both in control and after PTS (Figure 6A). This was also supported by the very low co-localization coefficients of SIRT2 with the astrocyte marker GFAP: about 0.10 (Figure 6B). This is consistent with the literature data (Maxwell et al., 2011; Krey et al., 2015; Xie et al., 2017; Demyanenko et al., 2020).

### Apoptosis

**FIGURE 7:**
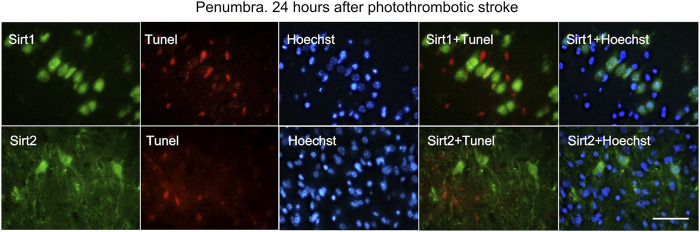
Immunofluorescence of SIRT1, SIRT2, TUNEL-positive apoptotic cells, nuclear chromatin marker Hoechst 33342, and image overlay in the penumbra 24 h after photothrombotic stroke in the rat cerebral cortex. The scale bar is 100 μm. Cells containing SIRT1 or SIRT2 (green) do not co-localize with the TUNEL-positive apoptotic cells (red).

Double fluorescent staining of penumbra tissue sections with antibodies against SIRT1 and SIRT2 and an apoptosis marker TUNEL (Figure 7) showed that 24 h after PTS, SIRT1 and SIRT2 did not co-localize with the nuclei of apoptotic cells, i.e., did not participate in PTS-induced apoptosis.

### Inhibitors

**FIGURE 8:**
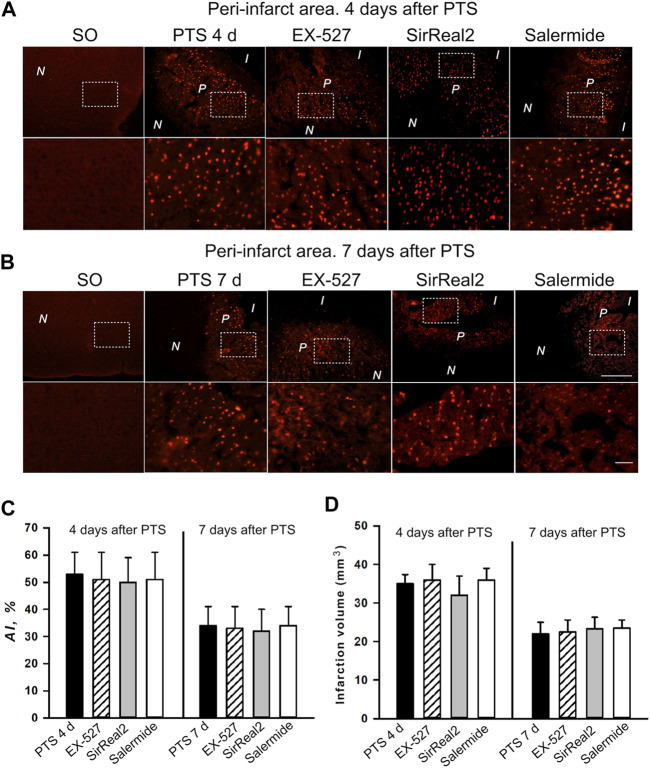
Apoptosis in the PTS-induced peri-infarct area in the mouse cerebral cortex. **(A,B)** Typical images of cortical regions stained with TUNEL (red fluorescence) at 4 days **(A)** and 7 days **(B)** after PTS. Control: sham-operated animals (SO). Experimental groups: The cerebral cortex of mice injected by various inhibitors. Inhibitors: EX-527, a selective SIRT1 inhibitor; SirReal2, a selective SIRT2 inhibitor; salermide, a nonspecific inhibitor of SIRT1 and SIRT2. Scale bar 200 μm **(C)** Changes in the apoptotic index (AI, %) in the mice of experimental groups on day 4 and 7 after photothrombotic stroke and introduction of various inhibitors. **(D)** The effects of SIRT1 and SIRT2 inhibitors on the infarction core volume in the mouse brain at 4 and 7 days after photothrombotic stroke. Mean values of the infarction core volume (mm^3^) in the control group (PTS without inhibitors) and in the experimental groups (administration of inhibitors). Scale bar 1 cm. One Way ANOVA; M ± SEM; *n* = 7–10. *p < 0.05 compared with the PTS in the absence of inhibitors.

Data on the participation of epigenetic proteins in the reactions of penumbra cells prompts the study of their inhibitors’ effects to confirm the role of these proteins in the penumbra cells’ response to ischemic brain damage, as well as to identify potential neuroprotectors that can protect brain cells from the consequences of ischemic stroke. We studied the effect of SIRT1 and SIRT2 inhibitors on the apoptosis of peri-infarct area cells and the infarction volume in the brain of mice, developing 4 or 7 days after photothrombotic stroke (Figure 8). For this purpose, the mice were injected with EX-527, a selective SIRT1 inhibitor; SirReal2, a selective SIRT2 inhibitor or salermide, a nonspecific inhibitor of SIRT1 and SIRT2.

In the sections of the cerebral cortex subjected to photothrombotic stroke, the nuclei of apoptotic cells are localized in a band about 1–1.5 mm wide. To the left and right of this band, where the infarction core and normal tissue are located, respectively, apoptosis is not observed. The infarction volume and the Apoptotic index (AI), with the introduction of all the studied inhibitors of sirtuins practically, did not differ from the control (PTS without inhibitors) (Figure 8).

## Discussion

SIRT1 has been shown to play significant roles in health and disease.

SIRT1 is widely expressed in the glial cells, neural stem cells, microglia, neurons and astrocytes of adult brain (Koronowski and Perez-pinzon, 2015; Demyanenko et al., 2020), our data confirm this. Regarding its role in cardiovascular diseases (CVDs) and neurobiology, previous studies provided evidence that SIRT1 reduction links vascular senescence and inflammation to abdominal aortic aneurysms (AAA) and that SIRT1 in vascular smooth muscle cells provides a therapeutic target for the prevention of AAA formation (Chen et al., 2016). It was shown earlier that SIRT1 localized mainly in the neuronal nuclei (Koronowski and Perez-pinzon, 2015). In our study, the expression of SIRT1 also increased in astrocytes on the first day after PTS. The physiological effect of this increased expression in astrocytes remains to be seen. Recently, the SIRT1 activator resveratrol has been shown to reduce neurotoxicity by altering glial activity and promote normal astrocyte function, which release energy, make ATP available to neurons, and decrease reactive oxygen species (Ghazavi et al., 2020). SIRT1 levels in the penumbra cells were significantly decreased in the nuclei and increased in the cytoplasm after PTS. This redistribution demonstrated the involvement of SIRT1 in the rat’s cerebral cortex response to photothrombotic impact in the acute neurodegeneration period. The expression and localization of SIRT1 in neurons and astrocytes of the cerebral cortex of mice during the recovery period after a PTS have been recently reported (Demyanenko et al., 2020). An increase in the level of SIRT1 expression up to 14 days after PTS was observed in both neurons and astrocytes of the mouse cerebral cortex, along with its redistribution to the cytoplasm. Interestingly, the nuclear form of SIRT1 suppresses cell apoptosis (Tanno et al., 2007), while the cytoplasmic form enhances it (Jin et al., 2007). However, we were unable to detect SIRT1 expression in apoptotic penumbra cells 24 h after PTS, as well as 7 days after PTS (Demyanenko et al., 2020).

In addition, in our study, the inability of EX-527, a selective SIRT1 inhibitor, to block PTS-induced apoptosis in the penumbra and reduce infarction volume revealed that SIRT1 is not involved in the mouse cerebral cortex response to photothrombotic stroke. The blood-brain barrier (BBB) penetrability of EX-527 has not yet been well established, which could be a limiting factor to SIRT1 inhibition in the CNS. Taking into account that EX-527 has demonstrated a partial SIRT1 inhibition in the brain (Bonomi et al., 2018). We can assume that the inability of EX-527 to change the level of apoptosis and the volume of damage in the peri-infarct area indicates the inability of the cytoplasmic form of SIRT1 to activate apoptosis. However, an increase in protein expression on the first day after PTS indicates its involvement in the response to PTS.

Furthermore, microinjection of EX-527 has recently been shown to reduce the volume of ischemic brain infarction and improve survival, but does not reduce the neurological deficits associated with stroke (Nikseresht et al., 2019). Moreover, the effect of EX-527 was associated with its ability to remove ischemia-induced impairment of the activity of metabolic enzymes associated with necroptosis. However, inhibition of necroptosis by Nec-1 microinjection did not affect Sirt1 expression levels.

Understanding the reason for the increased expression of SIRT1 in the acute period after PTS will be the subject of our further studies. Possibly, activation of Sirt1 on the first day after PTS is associated with an increase in the permeability of the blood-brain barrier (BBB), since the PTS model is characterized by a significant BBB impairment (Uzdensky, 2018), and suppression of Sirt1 by siRNA or salermide significantly reduces the permeability of BBB (Chen et al., 2018).

SIRT2 is also widespread in the brain, especially in oligodendrocytes, but not in astrocytes or microglia. SIRT2 levels are low in neurons (Maxwell et al., 2011; Krey et al., 2015). Expression of SIRT2 in the PTS-induced penumbra astrocytes was not detected. A significant increase in its levels in the cytoplasmic fraction of the penumbra was noted 24 h after PTS and remained increased up to 3 days after PTS (Demyanenko et al., 2020). SIRT2 has also been detected in the nuclei of the penumbra neurons. Similar findings indicate that SIRT2 is upregulated in ischemic neurons in the transient middle cerebral artery occlusion (tMCAO) mouse model and the oxygen-glucose deprivation cell model (Xie et al., 2017).

SIRT2 has been found to be associated with many neurological diseases, including ischemic stroke. After a stroke, the SIRT2 protein is significantly expressed in myelin-rich brain regions, and Sirt2 knockout mice displayed reduced neurological impairments in MCAO models with various occlusion times (Krey et al., 2015). SIRT2 has been also proved to be associated with cardiovascular diseases. Loss of SIRT2 reduces AMP-activated protein kinase (AMPK) activation, promotes aging-related and Ang II-induced cardiac hypertrophy, which indicates that SIRT2 could be a potential target for therapeutic interventions in aging- and stress-induced cardiac hypertrophy (Tang et al., 2017). In addition, SIRT2 could serve as a potential target to enhance remyelination during ageing, since it has been involved in rescuing the aged oligodendrocyte progenitor cells (OPCs) differentiation potential to levels comparable to young age (Ma et al., 2022). In this study, applying SirReal2 (Sirt2-selective inhibitor) did not change the infarction volume or the apoptotic index, compared to the control samples, which indicates that SIRT2 is also not involved in proapoptotic processes in the acute phase after PTS. It was previously shown that another SIRT2 inhibitor, AK-7, which is permeable to the brain, also showed no positive effects in the model of ischemia/reperfusion induced by carotid artery occlusion in mice (Chen et al., 2015). Although in the mouse model of middle cerebral artery occlusion (MCAO) it was shown that the AK-7 inhibitor significantly reduced the volume of infarction and promoted effective restoration of neurological function in mice (Wu et al., 2018). The authors suggest that one of the mechanisms of this effect may be phosphorylation of P38 after ischemic reperfusion injury, which can directly or indirectly phosphorylate Sirt2 and reduce its catalytic activity, thereby reducing ischemic damage by slowing cholesterol biosynthesis (Wu et al., 2018).

We studied also the effects of salermide, a nonspecific inhibitor of SIRT1 and SIRT2. As for other chosen inhibitors in this article, salermide administration did not demonstrate any difference from control samples (without inhibitors).

Thus, the growth of SIRT1 and SIRT2 levels in neurons and of SIRT1 in astrocytes of penumbra, as well as the redistribution of SIRT1 to neuronal cytoplasm, undoubtedly indicate the involvement of these sirtuins in the response of brain cells to ischemia. However, the performed inhibitor analysis has not revealed involvement of these proteins in the regulation of penumbra cell apoptosis in acute period after PTS. Perhaps, the function of SIRT1 and SIRT2 in the ischemic brain is associated with another form of cell death, for example, necroptosis. Our studies also show that activation of SIRT1 during the acute period of stroke does not have an unconditioned neuroprotective effect, while SIRT2 is associated with damage to penumbra cells. Studies of the relationship between SIRT1 expression and activity and NAD^+^ production in penumbra cells are needed.

Furthermore, the forkhead box O3a transcription factor (FoxO3a) is the substrate of SIRT1, SIRT2, and mitochondrial SIRT3. SIRT1 activity suppresses FoxO3a during ischemic stroke, which leads to the suppression of several proapoptotic factors such as p53, TNF-related apoptosis-inducing ligand (TRAIL), Fas ligand (FasL), Bcl-2-like protein11 (Bim), and activation of genes for antioxidant defense enzymes catalase and mitochondrial manganese-dependent superoxide dismutase (MnSOD), which protects neurons from apoptosis (She et al., 2017). However, SIRT2 activates FoxO3a by deacetylating it, which promotes the activation of the proapoptotic pathways AKT/FoxO3a and JNK (She et al., 2018). In cerebral ischemia, deacetylation of FoxO3a by SIRT3 causes the translocation of FoxO3a into the nucleus and enhancement of FoxO3a-dependent antioxidant protection associated with the activation of catalase and superoxide dismutase 2 (SOD2) (Yin et al., 2015). Interestingly, ischemic-reperfusion injury to the myocardium in rats induces FoxO3a activation by SIRT3. This enhances mitophagy through activation of the PINK1-Parkin pathway (Das et al., 2014). In turn, the SIRT1 activator resveratrol enhances the SIRT1/SIRT2-Foxo3a-PINK1-Parkin signaling cascade, which leads to cardioprotection (Das et al., 2014). More research is needed to elucidate the mechanisms of the synergistic effects of SIRT1, SIRT2, and SIRT3 on the protection and death of penumbra cells in stroke.

Moreover, disruption of mitochondrial homeostasis and cellular energy is one of the main reasons for the death of brain cells during ischemic-reperfusion injury, and therapy strategies associated with maintaining the normal functioning of mitochondria during ischemia are very promising (Ham and Raju, 2017; She et al., 2017; Khoury et al., 2018).

Most likely, the function of SIRT1 and SIRT2 in the ischemic brain is much more complex than just proapoptotic or antiapoptotic, cellular and intracellular localization of enzymes, as well as intracellular signaling pathways, regulating SIRT1 and SIRT2, but also related to the distance from the infarct core, and the degree of damage in mitochondria of brain cells after ischemia.

It should be noted that the model of photothrombosis used in our study has some limitations. This is a model of occlusion of small rather than large vessels of the brain, which makes it possible to study the molecular mechanisms of ischemic damage, rather than the mechanisms of ischemia-reperfusion damage to cells. This reduces the efficiency of this stroke model in the search for neuroprotectors capable of preserving penumbra cells after reperfusion (Uzdensky, 2018).

## Data Availability

The original contributions presented in the study are included in the article/Supplementary Material, further inquiries can be directed to the corresponding author.
