# Supplementary material for: Sirtuins 1 and 2 in the Acute Period After Photothrombotic Stroke: Expression, Localization and Involvement in Apoptosis
Source: Front Physiol. 2022 Apr 27;13:782684. doi: 10.3389/fphys.2022.782684 (PMC9092253; doi:10.3389/fphys.2022.782684)
Supplement: Supplementary file 1 [file Image1.pdf]

Supplementary Material

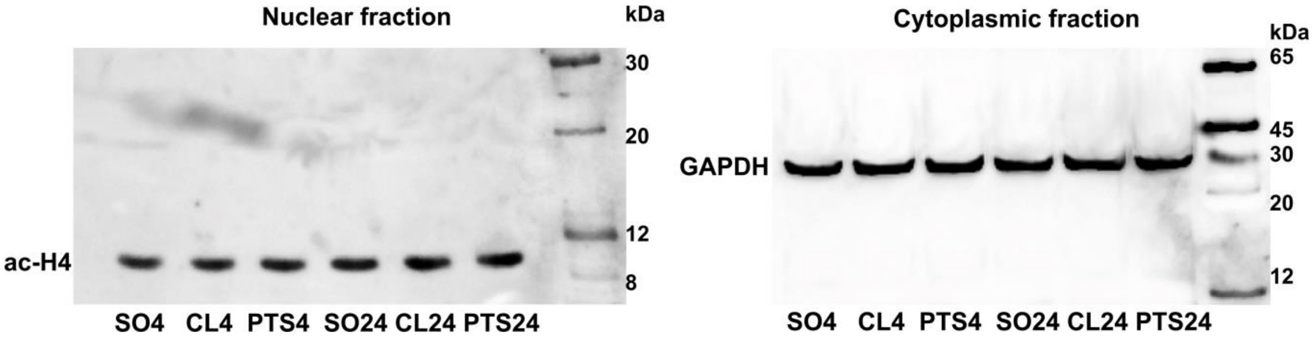

Supplementary Figure 1. Evaluation of the purity of the obtained nuclear and cytoplasmic fractions by Western blotting of penumbra tissue 4 h (PTS4) and 24 h (PTS24) after photothrombotic stroke in rats; cerebral cortex of the intact contralateral hemisphere (CL4 and CL24), as well as the cerebral cortex of sham operated animals 4 h (SO4) and 24 h (SO24) after irradiation. Acetylated histone H4 (ac-H4) protein was used as a nuclear fraction marker. We used Antiacetyl-Histone H4 produced in rabbits (# 06-866, Merck) at a dilution of 1:500. The protein glyceraldehyde-3-phosphate dehydrogenase (GAPDH) was used as a marker of the cytoplasmic fraction. We used the Anti-GAPDH antibody produced in rabbits (G9545, Sigma Aldrich) at a dilution of 1:1000.
